# AmalgamScope: Merging Annotations Data across the Human Genome

**DOI:** 10.1155/2014/893501

**Published:** 2014-05-20

**Authors:** Georgia Tsiliki, Konstantinos Tsaramirsis, Sophia Kossida

**Affiliations:** ^1^Bioinformatics and Medical Informatics Team, Biomedical Research Foundation, Academy of Athens, 115 27 Athens, Greece; ^2^Henley Business School, Business Informatics, University of Reading, Whiteknights, Reading RG6 6UD, Uk

## Abstract

The past years have shown an enormous advancement in sequencing and array-based technologies, producing supplementary or alternative views of the genome stored in various formats and databases. Their sheer volume and different data scope pose a challenge to jointly visualize and integrate diverse data types. We present AmalgamScope a new interactive software tool focusing on assisting scientists with the annotation of the human genome and particularly the integration of the annotation files from multiple data types, using gene identifiers and genomic coordinates. Supported platforms include next-generation sequencing and microarray technologies. The available features of AmalgamScope range from the annotation of diverse data types across the human genome to integration of the data based on the annotational information and visualization of the merged files within chromosomal regions or the whole genome. Additionally, users can define custom transcriptome library files for any species and use the file exchanging distant server options of the tool.

## 1. Background

A major advancement in the field of biomedical research is that currently researchers analyze multiple types of data, such as expression profiling, whole genome sequencing and other high-throughput experiments, which correspond to complementary views of a single organism [1]. Those diverse data types improve our ability to detect gene sets associated with a phenotype of interest. For instance, cancer genomes contain point mutations, methylation abnormalities, copy number, and expression changes not seen in normal tissues [2]. To fully comprehend those data, often called “omics” data, one needs to consult publicly available databases provided by major bioinformatics organizations, such as the National Center for Biotechnology Information (NCBI; http://www.ncbi.nlm.nih.gov/) and the European Bioinformatics Institute (EBI; http://www.ebi.ac.uk/). Their sheer volume accompanied with auxiliary information, justify for novel tools that are able to flexibly scale, integrate, and jointly visualize them.

Along these lines, a number of efforts have been established which differ on the volume, type, and scope of data considered. Well-known visualization tools, such as the Ensembl genome browser [3], the University of California Santa Cruz (UCSC) genome browser [4], and NCBI's MapViewer [5], are built on top of the corresponding online public databases and for that reason provide access to a broad range of annotational information for a wide variety of organisms and different genome assemblies. Many stand alone software exist which retrieve information from the above and other databases but specialize in producing summary and visualization results for specific species or data types. Recently produced visualization software mostly explore next-generation sequencing (NGS) data alongside extra annotation from a reference genome, for instance, the MapView [6], the interactive GenomeView [7], AnyExpress [8], and the MGAViewer [9] software which offers visualization options in metagenomics studies. Representative examples of tools that focus on merging information from different data studies are the generic genome browser (GBrowse) [10] and its web server WebGBrowse [11] which allows users to upload their own genomic data for display. AnyExpress [8] accepts as input aligned data, removes undesirable probes, such as sequence repeats, and generates a target-by-sample text file. OmicsBrowse [12] connects different genomes and evolutional correspondences among multiple species derived from multiple data-servers; it displays both genetic maps and genomic annotations within wide chromosomal intervals and assists the user to select candidate genes by filtering their annotations or associated documents against user-specified keywords or ontology terms. Nevertheless, the above software accepts raw data or concentrates on analyzing user-defined chromosomal intervals and merges information from various data sources, rather than exploring data from whole array data.

The integrative genomics viewer (IGV) [13] is well suited for genome-wide exploration of NGS data. Particularly, the IGV tool has the ability to display data and dynamically group samples based on metadata supplied, giving particular emphasis on the data scaling option provided to the user. Additionally, GeneWeaver [14] is a curated repository of genomic experiments which allows the user to perform integrative functional genomics in combination with the incorporated data. When it comes to merging the data in an annotational level, in order to computationaly analyze them, it is crucial to be able to extract the data mapping and the summary information that is graphically displayed. Existing tools are not flexible enough to support custom changes, or updated versions of platform databases, whereas such options are available through less user-friendly programs. For example, the Galaxy platform [15] and also the statistical computing environment R (http://www.r-project.org/) via Bioconductor (http://www.bioconductor.org/) provide extensive annotation resources (e.g., AnnotationDBi) as well as merging capabilities (e.g., GenomicRanges, GenomicFeatures packages).

To address these issues, we present a processs which automates the matching of NGS and microarray data by scaling them to genes and also allows users to customize their database files in order to include only gene sets capturing established knowledge about biological processes and pathways. AmalgamScope (Amalgam) is an online stand alone tool with a user-friendly graphical interface which allows merging of large diverse datasets on an annotational level, as well as offering an interactive graphical user interface.

Amalgam aims to create a map of the human genome based on the annotation files supplied by the user, primarily focusing on the genomic context, whilst augmenting the available information from well-known databases. The user follows a stepwise procedure to derive the merged data, namely, a knowledgable summary of the human data uploaded, which can then be used for complementing computational analysis. The suggested pipeline greatly reduces the time, expertise, and error involved in assembling a common vocabulary for diverse data, offering the baseline for the development of analytical integration methodologies and also a common platform for reproducible exploration of the transcriptome. An important advantage of the tool is the option to upload customized input files for merging and placing equal weight on each dataset, and in a similar way, the integration of files can be based on customized library files of any species. Data supported are derived by sequencing and microarray technologies and currently include RNA sequencing (RNA-Seq), microarray gene expression, copy number variants (CNVs), and DNA methylation data, though it is possible to import any data files with entities that can be assigned to either genes or chromosomal locations in the human genome. Additionally, a special feature of Amalgam is the option to manage input or output from distant hosting servers.

## 2. Implementation

To merge the data uploaded, we consider a common denominator, that is, an entity which can serve as an intermediate link between the various data types. Since gene names and chromosomal locations are reported in most of the data produced by microarray and sequencing technologies, we have considered both entities as the default “regional units” where annotations from all datasets would be translated into. The user can choose a different regional unit as long as that is present in all loaded datasets and define it via the “Settings” option. Amalgam aims to identify the wider, in terms of base pairs, non-overlapping regional units across datasets which include at least one of the entities supplied. For that reason, it identifies a list of unique genomic features across datasets and by that constructs a vocabulary of regional units, where the merging scheme is based on and all visualization options are later displayed in. The output is an integrated view of the data mapped onto the human genome, along with genomic annotations from public databases.

### 2.1. Translating and Merging

By uploading the data files, the user is prompted to choose between local and web annotation retrievals. The local download retrieves data from three local repositories downloaded from NCBI, Ensembl (http://www.ensembl.org/index.html), and UCSC Browser (http://genome.ucsc.edu/) databases. The local library files include gene names and synonyms, gene identifiers, and chromosomal locations in base pairs. The web annotation retrieval is comparatively time-consuming, as it directly connects with the above databases to retrieve up-to-date and possibly missing information. Additionally, the user can choose to upload customized library files and proceed with merging the data.

**Figure 1:**
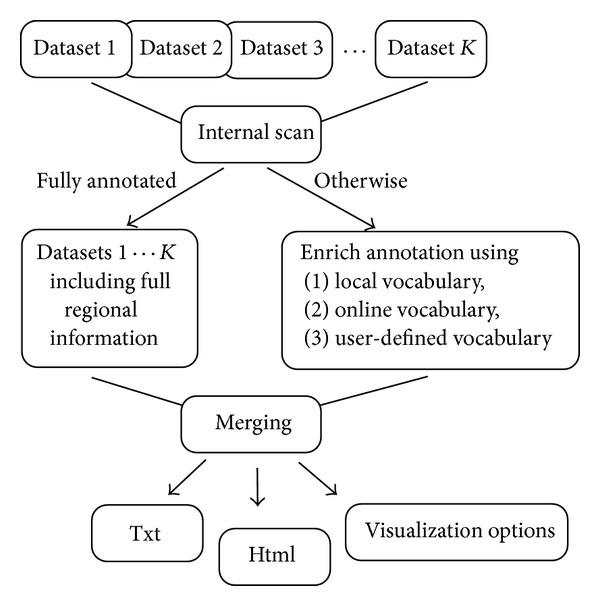
Schematic view of the procedure followed by Amalgam. Input files, Datasets 1 ⋯ *K*, are scanned to ensure that all entries include regional information. If that is not the case a reverse annotation procedure is followed to produce the unique annotational vocabulary. After merging the files, annotational information is available in Txt and Html formats, along with visualization options.

Upon completion of the merging procedure, the user can browse and download the merged tabulated files as formated text or HTML file format. The latter includes active links to the above mentioned three databases. The results are organized based on the identified vocabulary of regional units and the data types considered. If the input data files include extra information, such as metadata or data values, then those will be also available in the merged files. Compressed formats can be emailed to a user specified address, enabling a quick data exchange (backup) of information which could be reused as input to avoid repetition of the same analysis. In Figure 1 we show a schematic representation of how *K* different datasets (Dataset 1 ⋯ *K*) are processed using Amalgam to produce a merged annotation file mapped onto the human genome. The user can download the merged file or alternatively launch the graphical interface options.

### 2.2. Visualization Options

**Figure 2:**
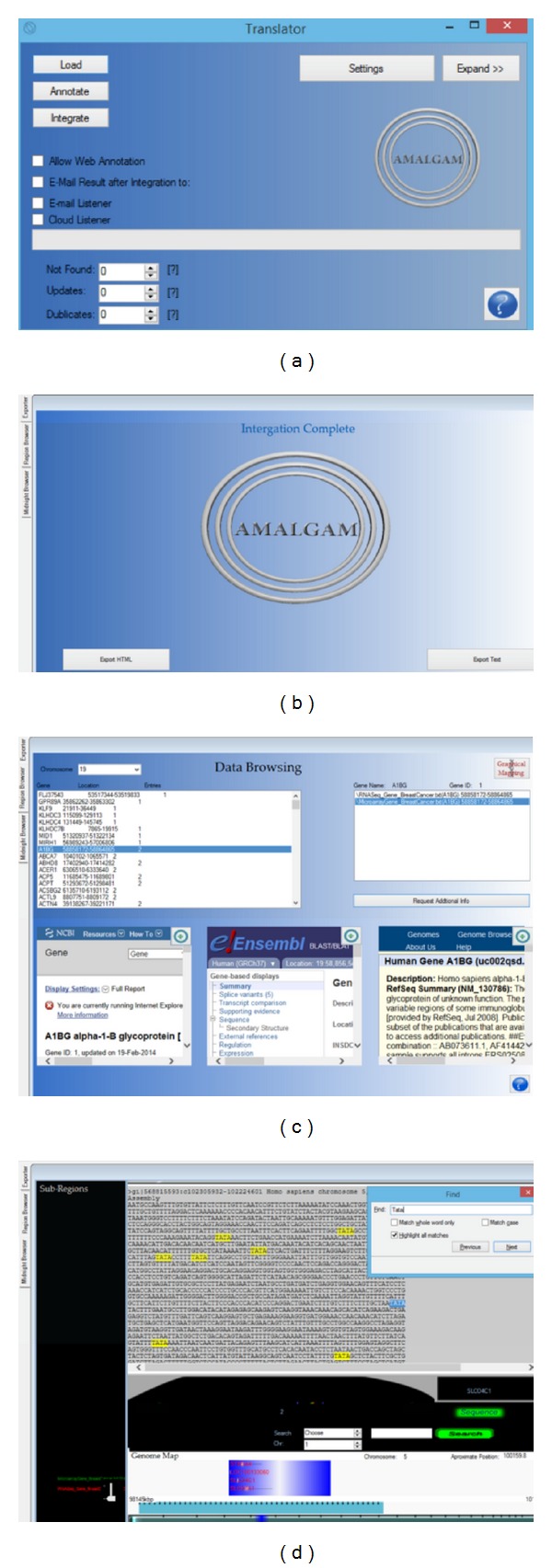
Screenshots of Amalgam outlining the key features of the application. The “Translator” window (a) awaits for input files allowing users to upload text annotation or data files. When merging is complete, the output files can be downloaded by the “Integrator” tab page (b). The output merged files can be displayed in the two browsing windows. Particularly, genomic features, such as gene names and chromosomal positions, are extracted from the text files and are displayed in the “RegionBrowser” tab page (c). For each identified region, gene related information can be displayed from well-known public databases. In the “Midnight Viewer” tab page (d), the merged data are displayed on chromosomal or genome maps.

Amalgam offers two visualization options for an intuitive real-time exploration of the merged data, called “Region Browser” and “Midnight Browser,” respectively. The first visualization tab page displays the identified list of regional units and their summaries; upon clicking a region, region-specific details appear via the above-mentioned databases. The second visualization window provides chromosome and human genome maps of the integrated data, where the user can launch a genome-wide exploration to see the identified regional units together with their individual annotational information. Navigation through the merged data ranges from whole genome to base pair level.  Figure 2 shows the processing windows of Amalgam. Particularly, in Figure 2(a) the window for uploading and translating the data, either locally or remotely, is shown, whereas Figure 2(b) shows the “Integrator” tab page as it appears when the mapping procedure is completed and the output file is available in Txt and Html formats. At the bottom of Figure 2, the Amalgam's integrated data visualization options are shown. In Figure 2(c) the merged files are presented in a list, given the regional units identified. For each regional unit, relative annotational information are supplied, whereas the user also has the option to further explore the information derived by NCBI, Ensembl, and UCSC databases.  Figure 2(d) shows the “Midnight Viewer” tab window, where we can observe the regional units vocabulary derived by diverse datasets (i.e., microarray gene expression, RNA gene expression, and CNV). A search by term option is offered, as well as the FASTA files of the genes found in the identified regional units.

### 2.3. Use Distant Server

A special feature of Amalgam is the option to manage input or output files from local exchange email clients or file hosting services. Amalgam uses an internal timer to receive input from distant email servers and cloud storage servers, “E-mail Listener” and “Cloud Listener” options, respectively, as shown in Figure 2(a). Every few seconds, feedback is requested from the server. If a new email has been received and it is qualified for processing, Amalgam downloads the attached files, processes them locally, and resends them using the given email server. Similarly, if new files have been uploaded on a given directory, Amalgam processes them and saves the output to the given output directory. The reason for using internet hosting or emailing services is to allow the users to access their results from any network device.

## 3. Results and Discussion

Amalgam was tested with publicly available data downloaded from The Cancer Genome Atlas (TCGA; http://cancergenome.nih.gov/) data portal, but can be easily applied to any datasets given that gene name or gene identification is included at the uploaded files or alternatively chromosome name along with chromosomal locations given in base pairs. Datasets can be loaded from local or remote sources, enabling users to merge their data with other publicly available data of interest. As a case study, we applied Amalgam to merge three publicly available human breast cancer samples downloaded from the TCGA data portal, that is, an Agilent gene expression microarray (G4502A) sample, an Illumina HiSeq RNA-Seq (V2), and a copy number variant data sample from Affymetrix Genome wide SNP 6.0 array. Amalgam finds the wider, in terms of base pairs, regional units for all data samples to constitute a unique genome vocabulary of non-overlapping intervals. Each regional unit originates from one of the three data samples and may include subregions, that is, entries from the remaining two data samples. Diverse data types are merged, placing equal weights to each dataset.

**Figure 3:**
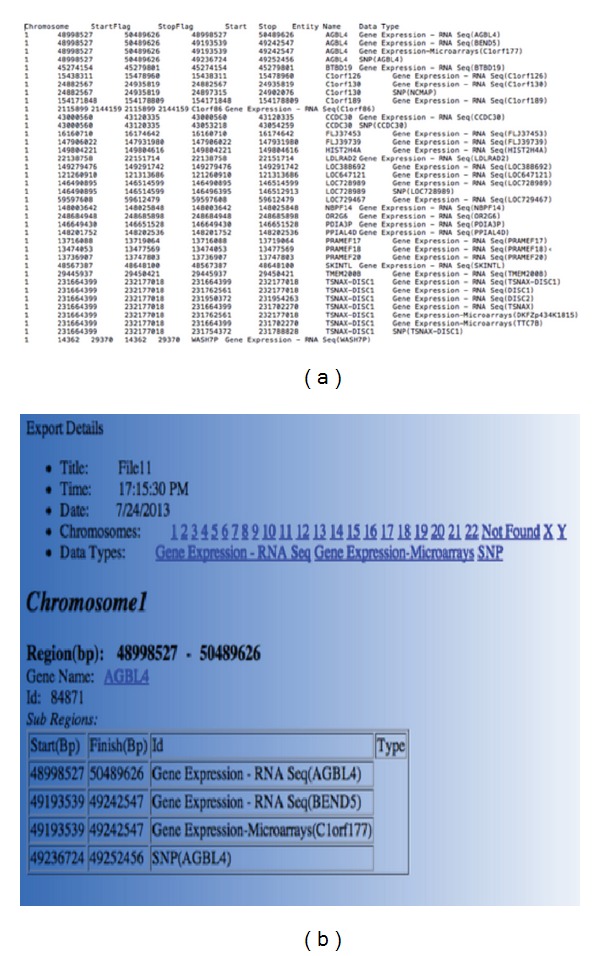
Txt and Html formats of the merged file are produced. Three breast cancer TCGA samples were uploaded and merged. Each sample is derived by a different platform, namely, Agilent gene expression microarray, Illumina RNA-Seq, and Affymetrix copy number variant data. (a) The tabulated merged file includes chromosomal id, start/end positions of the identified regional units (StartFlag, StopFlag), and the subregions included in the identified regions (Start, Stop), the region name (Entity name) and the data type that the region belongs to. (b) The same information as in (a) is displayed in Html format with active links to the NCBI database.

In Figure 3 we show the merged files produced from the three data samples uploaded. As can be seen from the Txt (Figure 3(a)) and Html (Figure 3(b)) exports produced, information for the regional units identified is given, as well as the subregions included in each regional unit. In the example presented 52,932 unique regions were identified, from which 343 entries did not map to gene or other regional information based on our local databases. Using the “Web Annotation” option, we identified 293 additional entries. This information can be also displayed using Amalgam's visualization browsers as shown in Figures 2(c) and 2(d). Reusability is ensured since the user can relaunch Amalgam and merge an output from a previous Amalgam run with additional input sample files.

Additionally, custom files can be uploaded, either as input or library files, enabling users to augment, merge, and visualize their data with publicly available data. This, also ensures that the tool is up-to-date at all times, since apart from hosting custom-made libraries, it is also able to house a compiler when running.

## 4. Conclusions

Integration and analysis of large diverse datasets is a promising field of ongoing research towards the understanding of the genome and its relation to human disease [2, 16]. However, merging diverse data types in the genome often becomes a time-consuming task due to the continuous improvement of the underling technologies and certain format incompatibilities. This calls for intuitive tools able to flexibly integrate multiple data types and produce a common vocabulary map of the human genome given the supplied data. We have developed AmalgamScope, a user-friendly stand-alone tool for merging annotation files of diverse genomic data types, including data values. Our software provides a flexible framework to summarize fragmented annotation information across data types, relative to information extracted from web-based annotation resources, providing a common platform for reproducible exploration of the transcriptome. The integrated annotation files can considerably assist further analysis for data integration or visualization.

To improve the management and storage of input/output files, an emailing exchange procedure is included together with a cloud utility; however, in the future we aim to further explore Amalgam's cloud capabilities, given the complexity and volume of the data. Currently, we are extending Amalgam in a distributed computing environment to accommodate larger studies.

## Availability and Requirements

*Project Name.* AmalgamScope.

*Project Home Page.* http://www.bioacademy.gr/bioinformatics/Amalgam/index.html.

*Operating System.* Microsoft Windows. Programming language: C sharp. License: Freeware.
